# Nomenclature and listing of celiac disease relevant gluten T-cell epitopes restricted by HLA-DQ molecules

**DOI:** 10.1007/s00251-012-0599-z

**Published:** 2012-02-10

**Authors:** Ludvig M. Sollid, Shuo-Wang Qiao, Robert P. Anderson, Carmen Gianfrani, Frits Koning

**Affiliations:** 1 Centre for Immune Regulation and Department of Immunology, University of Oslo and Oslo University Hospital - Rikshospitalet, Oslo, Norway; 2 Walter and Eliza Hall Institute of Medical Research, Parkville, Victoria, Australia; 3 Institute of Food Science, National Research Council, Avellino, Italy; 4 Department of Immunohematology and Blood Transfusion, Leiden University Medical Center, Leiden, The Netherlands

**Keywords:** Celiac disease, Gluten, T cell, Epitopes, HLA-DQ2, HLA-DQ8

## Abstract

Celiac disease is caused by an abnormal intestinal T-cell response to gluten proteins of wheat, barley and rye. Over the last few years, a number of gluten T-cell epitopes restricted by celiac disease associated HLA-DQ molecules have been characterized. In this work, we give an overview of these epitopes and suggest a comprehensive, new nomenclature.

Celiac disease (CD) is caused by an abnormal intestinal immune response to proline and glutamine-rich wheat gluten proteins and to similar proteins in barley and rye (Green and Cellier 2007). Oat is generally considered safe for consumption by CD patients (Garsed and Scott 2007), although some patients appear to be sensitive to oat as well (Lundin et al. 2003). The only established treatment for the disease is a lifelong gluten exclusion diet.

CD has a strong HLA association. The most prominent association is with HLA-DQ2.5 (DQA1*05:01, DQB1*02:01) (Table 1). In individuals who carry the DR3DQ2 haplotype, this molecule is encoded by DQA1 and DQB1 alleles located on the same chromosome (in *cis* configuration), whereas in individuals who are DR5DQ7/DR7DQ2 heterozygous it is encoded by alleles located on opposite chromosomes (*trans* configuration) (Sollid et al. 1989). Most of the remaining patients carry DR4DQ8 haplotypes, and in these patients it is the DQ8 molecule encoded by DQA1*03, DQB1*03:02 that is involved in the pathogenesis (Lundin et al. 1994). In the small remaining population of CD patients that are neither DQ2.5 or DQ8, the patients typically express HLA-DQ molecules that contain ‘half’ of DQ2.5 molecule as they are either DQ2.2 (DQA1*02:01, DQB1*02:01) or DQ7.5 (DQA1*05, DQB1*03:01) (Karell et al. 2003). As both the DQA1 and DQB1 loci are polymorphic, unique HLA-DQ molecules can be encoded in *trans* configuration. Examples of such molecules are DQ2.3 (DQA1*03, DQB1*02:01) and DQ8.5 (DQA1*05, DQB1*03:02) found in DR3DQ2/DR4DQ8 heterozygous individuals (Table 1).

**Table 1:** Description and naming of HLA-DQ molecules that are associated with celiac disease and which are used as antigen presenting elements for CD4^+^ T cells of celiac disease patients

	Encoded by		Risk for celiac disease	Expression in *cis* or *trans* position	Part of common *cis* haplotype
HLA-DQ molecule	DQA1*	DQB1*			
HLA-DQ2.5	05	02	High	*cis, trans*	DR3DQ2
HLA-DQ2.2	02	02	Low	*cis, (trans)*	DR7DQ2
HLA-DQ2.3	03	02	Likely low^a^	*trans, (cis)* ^b^	
HLA-DQ7.5	05	03:01	Very low	*cis, (trans)*	DR5DQ7
HLA-DQ8	03	03:02	Low	*cis*	DR4DQ8
HLA-DQ8.5	05	03:02	Likely low^a^	*trans, (cis)* ^b^	

^a^Risk for celiac disease has not been established in population studies

^b^Molecule can also be encoded in *cis* on some rare haplotypes

CD4^+^ T cells of CD patients (Lundin et al. 1993), but not healthy subjects (Molberg et al. 1997), recognize gluten peptides when presented by disease associated HLA-DQ molecules. This was first shown for DQ2.5 and DQ8 (Lundin et al. 1994), and recently it was demonstrated that DQ2.2 patients (Bodd et al. 2012) and a patient who carries DQ8.5 in a rare *cis* configuration (Kooy-Winkelaar et al. 2011) also have gluten-reactive T cells in the intestinal mucosa. Gluten-reactive T cells can readily be established from intestinal biopsies cultured in vitro (Camarca et al. 2009; Lundin et al. 1993, 1994; Tye-Din et al. 2010; Vader et al. 2002b; van de Wal et al. 1998b). T cells recognizing the same gluten epitopes are normally not detected in the peripheral blood (Anderson et al. 2000), but can be found in the blood of treated CD patients on day 6 after a 3-day oral gluten challenge (Anderson et al. 2000, 2005; Ráki et al. 2007).

HLA is the most important genetic factor in CD, and carriage of certain HLA alleles is a necessary, but not sufficient, factor for disease development (Sollid 2002). The other factors required for disease development are non-HLA genes, of which 39 loci have been identified so far (Trynka et al. 2011), and possibly environmental factors other than gluten. To note, mice transgenic for HLA-DQ2.5 and gluten specific T-cell receptors do not develop a CD-like enteropathy (de Kauwe et al. 2009; Du Pre et al. 2011). The reason why these mice do not develop enteropathy may relate to fundamental differences in the gut physiology between mouse and man, and to the lack of appropriate non-MHC genes in the mouse strains tested that parallel the non-HLA susceptibility genes of CD patients.

The differential risk of DQ2.5 and DQ2.2 is linked with the T-cell response to gluten. It has been demonstrated that DQ2.5 binds a larger gluten peptide repertoire compared to DQ2.2 (Vader et al. 2003b). Further, gluten T-cell epitopes form stable complexes with DQ2.5, and the increased risk of DQ2.5 over DQ2.2 correlates with a different ability of the two HLA molecules to form stable complexes with many gluten peptides (Fallang et al. 2009).

Characteristically, gluten-reactive T cells of CD patients recognize their antigenic peptides much better when specific glutamine residues are converted to glutamate by the enzyme transglutaminase 2 (TG2) (Molberg et al. 1998; van de Wal et al. 1998a). Deamidated gluten peptides bind with increased affinity to DQ2.5 and DQ8 (Arentz-Hansen et al. 2000; Camarca et al. 2009; Henderson et al. 2007; Kim et al. 2004; Moustakas et al. 2000; Quarsten et al. 1999), and the rate of dissociation of deamidated gluten peptides from DQ2.5 has been shown to be substantially slower than for their native counterparts (Xia et al. 2005). The ability to form stable peptide–MHC complexes again seems to be a key factor for the initiation of the anti-gluten T-cell response.

Gluten is defined as the cohesive mass that remains when dough is washed to remove starch (Shewry et al. 1992). Traditionally and strictly speaking, gluten is a name of wheat proteins only, but gluten is now increasingly used as a term to denote proline- and glutamine-rich proteins of wheat, barley, rye and oat. In wheat, gluten consists of the gliadin and glutenin subcomponents. The gliadin proteins can be subdivided into α-, γ- and ω-gliadins, while the glutenin proteins can be subdivided into high molecular weight (HMW) and low molecular weight (LMW) subunits. Common bread wheat is a hexaploid species, and in addition some of the gluten protein encoding genes originate from duplicated loci. Thus, in a single wheat variety there exits up to several hundred different gluten proteins, many of which only differ by a few amino acids. The proline- and glutamine-rich proteins of barley, rye and oat are termed hordeins, secalins and avenins, respectively.

Given the heterogeneity of the wheat gluten proteins, it is no surprise that many distinct gliadin and glutenin derived T-cell epitopes exist (Table 2). T-cell epitopes derived from either α-, γ-, and ω-gliadins as well as from HMW and LMW glutenins have been reported (Arentz-Hansen et al. 2000; Sjöström et al. 1998; Vader et al. 2002b; van de Wal et al. 1998b). T-cell epitopes in both hordeins and secalins have been described and they are highly homologous to those found in wheat (Tye-Din et al. 2010; Vader et al. 2003a). The avenins of oat are more distinct, and although oat is considered safe for CD patients (Garsed and Scott 2007), some CD patients are clinically sensitive to oat (Lundin et al. 2003). Avenin specific as well as cross-reactive responses have been described (Arentz-Hansen et al. 2004; Vader et al. 2003a).

**Table 2:** List of celiac disease relevant T-cell epitopes recognized by CD4^+^ T cells

Epitope^a^	Previous names	Peptide-binding register^b^									Reference
		1	2	3	4	5	6	7	8	9	
DQ2.5 restricted epitopes											
DQ2.5-glia-α1a	DQ2-α-I, α9	P	F	P	Q	P	**E**	L	P	Y	(Arentz-Hansen et al. 2000)
DQ2.5-glia-α1b	DQ2-α-III	P	Y	P	Q	P	**E**	L	P	Y	(Arentz-Hansen et al. 2002)
DQ2.5-glia-α2	DQ2-α-II, α2	P	Q	P	**E**	L	P	Y	P	Q	(Arentz-Hansen et al. 2000)
DQ2.5-glia-α3	glia-α20	F	R	P	**E**	Q	P	Y	P	Q	(Vader et al. 2002b)
DQ2.5-glia-γ1	DQ2-γ-I	P	Q	Q	S	F	P	**E**	Q	Q	(Sjöström et al. 1998)
DQ2.5-glia-γ2	DQ2-γ-II, γ30	I	Q	P	**E**	Q	P	A	Q	L	(Qiao et al. 2005; Vader et al. 2002b)
DQ2.5-glia-γ3	DQ2-γ-III	Q	Q	P	**E**	Q	P	Y	P	Q	(Arentz-Hansen et al. 2002)
DQ2.5-glia-γ4a	DQ2-γ-IV	S	Q	P	**E**	Q	**E**	F	P	Q	(Arentz-Hansen et al. 2002)
DQ2.5-glia-γ4b	DQ2-γ-VIIc	P	Q	P	**E**	Q	**E**	F	P	Q	(Qiao et al. 2005)
DQ2.5-glia-γ4c	DQ2-γ-VIIa	Q	Q	P	**E**	Q	P	F	P	Q	(Arentz-Hansen et al. 2002)
DQ2.5-glia-γ4d	DQ2-γ-VIIb	P	Q	P	**E**	Q	P	F	C	Q	(Qiao, unpublished)
DQ2.5-glia-γ5	DQ2-γ-VI	Q	Q	P	F	P	**E**	Q	P	Q	(Arentz-Hansen et al. 2002)
DQ2.5-glia-ω1	DQ2-ω-I	P	F	P	Q	P	**E**	Q	P	F	(Tye-Din et al. 2010)
DQ2.5-glia-ω2	DQ2-ω-II	P	Q	P	**E**	Q	P	F	P	W	(Tye-Din et al. 2010)
DQ2.5-glut-L1	glutenin-17	P	F	S	**E**	Q	**E**	Q	P	V	(Vader et al. 2002b)
DQ2.5-glut-L2	glutenin-156	F	S	Q	Q	Q	**E**	S	P	F	(Stepniak et al. 2005; Vader et al. 2002b)
DQ2.5-hor-1	Hor-α9, Hα9	P	F	P	Q	P	**E**	Q	P	F	(Tye-Din et al. 2010; Vader et al. 2003a)
DQ2.5-hor-2	Hor-α2, Hα2	P	Q	P	**E**	Q	P	F	P	Q	(Vader et al. 2003a)
DQ2.5-hor-3	hor-I-DQ2	P	I	P	**E**	Q	P	Q	P	Y	(Tye-Din et al. 2010)
DQ2.5-sec-1	Sec-α9, Sα9	P	F	P	Q	P	**E**	Q	P	F	(Tye-Din et al. 2010; Vader et al. 2003a)
DQ2.5-sec-2	Sec-α2, Sα2	P	Q	P	**E**	Q	P	F	P	Q	(Vader et al. 2003a)
DQ2.5-ave-1a	Av-α9A	P	Y	P	E	Q	**E**	E	P	F	(Arentz-Hansen et al. 2004; Vader et al. 2003a)
DQ2.5-ave-1b	Av-α9B, 1490	P	Y	P	E	Q	**E**	Q	P	F	(Arentz-Hansen et al. 2004; Vader et al. 2003a)
DQ2.2 restricted epitopes											
DQ2.2-glut-L1	glutenin-17	P	F	S	**E**	Q	**E**	Q	P	V	(Bodd et al. 2012)
DQ8 restricted epitopes											
DQ8-glia-α1	DQ8-α-I	**E**	G	S	F	Q	P	S	Q	**E**	(van de Wal et al. 1998b)
DQ8-glia-γ1a	DQ8-γ-Ia	**E**	Q	P	Q	Q	P	F	P	Q	(Tollefsen et al. 2006)
DQ8-glia-γ1b	DQ8-γ-Ib	**E**	Q	P	Q	Q	P	Y	P	**E**	(Tollefsen et al. 2006)
DQ8-glut-H1	HMW-glutenin	Q	G	Y	Y	P	T	S	P	Q	(van de Wal et al. 1999)
DQ8.5 restricted epitopes											
DQ8.5-glia-α1	DQ8-α-I	**E**	G	S	F	Q	P	S	Q	**E**	(Kooy-Winkelaar et al. 2011)
DQ8.5-glia-γ1		P	Q	Q	S	F	P	**E**	Q	**E**	(Kooy-Winkelaar et al. 2011)
DQ8.5-glut-H1	HMW-glutenin	Q	G	Y	Y	P	T	S	P	Q	(Kooy-Winkelaar et al. 2011)

^a^In the epitope names, these short terms are used to denote the type of proteins that the epitopes derive from: ‘glia-α’ denotes α-gliadin, ‘glia-γ’ denotes γ-gliadin, ‘glia-ω’ denotes ω-gliadin, ‘glut-L’ denotes low molecular weight glutenin, ‘glut-H’ denotes high molecular weight glutenin, ‘hor’ denotes hordein, ‘sec’ denotes secalin and ‘ave’ denotes avenin

^b^Glutamate residues (E) formed by TG2-medited deamidation which are important for recognition by T cells are shown in bold. Additional glutamine residues also targeted by TG2 are underlined

There is at present no standard nomenclature for CD-relevant gluten epitopes. Here, we propose such a nomenclature based on the following three criteria:

1. Reactivity against the epitope must have been defined by at least one specific T-cell clone.
2. The HLA-restriction element involved must have been unequivocally defined.
3. The nine-amino acid core of the epitope must have been defined either by an analysis with truncated peptides and/or HLA-binding with lysine scan of the epitope or comparable approach.

Searching the literature using these criteria, we have compiled a list of epitopes (Table 2). This list includes sequences from α-gliadin, γ-gliadin, ω-gliadin, LMW- and HMW-glutenins, hordeins, secalins and avenins. To note, most gluten-reactive T cells have minimal epitopes longer than those listed in Table 2. This is because MHC class II restricted T-cell receptors usually are sensitive to a few residues flanking the nine-amino acid core region of the epitopes.

In Table 2, some sequences that previously were defined as individual epitopes have been grouped together as members of the same family. This pertains to the DQ2.5-glia-α1a and DQ2.5-glia-α1b as well as the DQ2.5-glia-γ4a, DQ2.5-glia-γ4b, DQ2.5-glia-γ4c and DQ2.5-glia-γ4d epitopes. The reason is that the sequences defining these epitopes are very similar, although there are occasionally T-cell clones that can distinguish between members of the same epitope family (Arentz-Hansen et al. 2002; Qiao et al. 2005). Most T-cell clones appear to cross-react between peptide sequences of the same family.

The nine-amino acid core sequences of some of the DQ2.5 restricted epitopes are identical, but because these epitopes derive from different cereal species they are still listed as unique epitopes. This applies to the DQ2.5-glia-ω1, DQ2.5-hor-1 and DQ2.5-sec-1 epitopes, as well as DQ2.5-hor-2 and DQ2.5-sec-2 epitopes. T-cell cross-reactivity between proteins of different species hence does often occur, but T cells reactive with these epitopes can also be species-specific as the T-cell receptors may be sensitive to unique residues flanking the nine-amino acid core region of the epitopes. In addition, there are epitopes, like DQ2.5-hor-3 (Tye-Din et al. 2010), that have sequences which are hordein or secalin specific and which elicit species specific T-cell responses.

The majority of gluten-reactive T cells generated from DQ2.5 positive CD patients can recognize their epitopes when confronted in vitro with antigen presenting cells expressing the closely related HLA-DQ2.2 molecule (DQA1*02:01, DQB1*02:01). Yet, DQ2.2 positive CD patients do not mount a T-cell response to the same gluten epitopes, but rather have responses to gluten peptides that bind stably to DQ2.2 (Bodd et al. 2012). When defining an epitope, it is thus important to assess and categorize the epitope only in the context of the HLA molecules that are expressed by the T-cell donor.

The selection of gluten T-cell epitopes is best understood in HLA-DQ2.5 positive CD patients, and is influenced by at least three factors: (a) resistance of the polypeptide sequence to proteolytic degradation, (b) specificity of TG2 and (c) HLA binding specificity. The proline-rich nature of gluten makes the gluten proteins resistant to proteolytic degradation in the gastrointestinal lumen, and long gluten peptide fragments ranging from 15 to 50 residues therefore survive in the small intestine (Shan et al. 2002). T-cell epitopes are often localized within such long fragments (Arentz-Hansen et al. 2002). The resulting proline and glutamine rich peptides are often good substrates for TG2 (Dørum et al. 2009, 2010; Fleckenstein et al. 2002; Vader et al. 2002a). Proline is influencing the specificity of TG2 as the enzyme typically recognizes glutamine residues in glutamine-X-proline sequences (Fleckenstein et al. 2002; Vader et al. 2002a). T-cell epitopes in their native form are usually very good substrates for TG2. TG2, being an important factor in the selection of T-cell epitopes, is demonstrated by the fact that TG2 is selectively targeting peptides which harbor T-cell epitopes from a digest of gluten with extreme complexity (Dørum et al. 2010). Finally, determinant selection by MHC is influencing repertoire of T-cell epitopes. In general, gluten peptides are poor binders to HLA class II molecules with the exception of HLA-DQ molecules associated with CD (Bergseng et al. 2008). Some gluten peptides also bind HLA-DR53 (Clot et al. 1999), although so far no T cells of celiac lesions recognizing these peptides have been described. The selective force of HLA is illustrated by the observation that the DQ2.5 and DQ8 epitopes generally come from non-overlapping sequences of gluten proteins (Tollefsen et al. 2006).

The glutamate introduced by TG2 is usually in position 4 (P4), P6 or P7 in HLA-DQ2.5 restricted epitopes and at position P1 and/or P9 in HLA-DQ8 restricted epitopes (Table 2). These glutamate residues serve as anchor residues important for binding of the peptides. Both HLA-DQ2.5 and DQ8 prefer negatively charged residues at these anchors sites. This positioning of deamidated glutamine residues is strongly related to the positioning of proline residues, which is particularly strict in the case of DQ2.5 epitopes, as DQ2.5 only accepts proline at certain position in the peptide binding groove (Kim et al. 2004). This results in a dominant presence of proline at P1, P6 and P8 and leads to the modification by TG2 of the glutamine residues at P4 and P6, respectively. Such positioning of proline residues is less strict in the case of the DQ8 epitopes. Although polyclonal T-cell responses to multiple T-cell epitopes are almost invariably found in CD patients, responses to the DQ2.5-glia-α1a, DQ2.5-glia-α1b, DQ2.5-glia-α2 epitopes, DQ2.5-glia-ω1, DQ2.5-glia-ω2, DQ2.5-hor-1 and DQ2-sec-1 are dominant in DQ2.5 positive patients (Arentz-Hansen et al. 2000; Camarca et al. 2009; Tollefsen et al. 2006; Tye-Din et al. 2010). In DQ8-positive patients, responses to the DQ8-glia-α1 epitope are most frequently found (Tollefsen et al. 2006; van de Wal et al. 1998b).

The list of gluten epitopes recognized by T cells of CD patients presented in Table 2 will be extended as new epitopes become known in the future. A dedicated website (http://www.isscd.org/EpitopeNomenclature) will update this list as more epitopes are identified.
